# CRISPR/Cas9 Gene Editing: An Unexplored Frontier for Forest Pathology

**DOI:** 10.3389/fpls.2020.01126

**Published:** 2020-07-22

**Authors:** Erika N. Dort, Philippe Tanguay, Richard C. Hamelin

**Affiliations:** ^1^ Department of Forest and Conservation Sciences, Faculty of Forestry, University of British Columbia, Vancouver, BC, Canada; ^2^ Laurentian Forestry Centre, Canadian Forest Service, Natural Resources Canada, Québec, QC, Canada; ^3^ Institut de Biologie Intégrative et des Systèmes (IBIS), Université Laval, Québec, QC, Canada; ^4^ Département des Sciences du bois et de la Forêt, Faculté de Foresterie et Géographie, Université Laval, Québec, QC, Canada

**Keywords:** forest diseases, tree disease resistance, filamentous pathogens, poplar rust, Dutch elm disease (DED), sudden oak death (SOD), white pine blister rust (WPBR)

## Abstract

CRISPR/Cas9 gene editing technology has taken the scientific community by storm since its development in 2012. First discovered in 1987, CRISPR/Cas systems act as an adaptive immune response in archaea and bacteria that defends against invading bacteriophages and plasmids. CRISPR/Cas9 gene editing technology modifies this immune response to function in eukaryotic cells as a highly specific, RNA-guided complex that can edit almost any genetic target. This technology has applications in all biological fields, including plant pathology. However, examples of its use in forest pathology are essentially nonexistent. The aim of this review is to give researchers a deeper understanding of the native CRISPR/Cas systems and how they were adapted into the CRISPR/Cas9 technology used today in plant pathology—this information is crucial for researchers aiming to use this technology in the pathosystems they study. We review the current applications of CRISPR/Cas9 in plant pathology and propose future directions for research in forest pathosystems where this technology is currently underutilized.

## Introduction

Developed in 2012, CRISPR/Cas9 gene editing is relatively new, but research using this technology has expanded rapidly in most scientific fields with 7,105 publications in 2019 alone (Clarivate Analytics, 2020). Even though human health and medicine are the most prolific fields, researchers in plant sciences are also starting to explore the applications of CRISPR/Cas9. The use of CRISPR/Cas9 in plant breeding has sparked interest in the field of plant pathology where disease resistant plant cultivars are becoming increasingly important. The applications of CRISPR/Cas9 technology in plant pathology have already been reviewed, especially in the context of agriculture (Langner et al., 2018; Makarova et al., 2018; Das et al., 2019; Muñoz et al., 2019). However, literature addressing its applications in forestry is lacking, and we believe this is because CRISPR/Cas9 is currently underutilized in this field. The purpose of this review is to fill this literature gap while giving forest pathologists a deeper understanding of CRISPR/Cas9 and its potential applications to better understand and manage tree diseases. We focus on how native CRISPR/Cas systems function as well as the mechanisms driving CRISPR/Cas9 gene editing technology as this information is crucial for implementation of this technology in forest pathosystems.

### What Is CRISPR/Cas? A Primer for Understanding CRISPR/Cas9 Gene Editing

**Figure 1:**
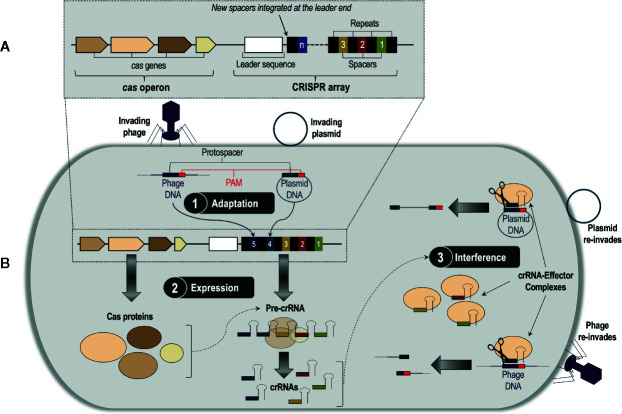
**(A)** A generalized CRISPR/Cas system consisting of a CRISPR array and a *cas* operon. The CRISPR array is composed of a series of identical repeat sequences interspaced with unique sequences called spacers, which are derived from the genetic material of invading bacteriophages and plasmids. On the 5′ end of the spacer/repeat locus is an intraspecies-conserved leader sequence likely involved in transcription of the CRISPR array. Flanking the CRISPR array is the *cas* operon, which contains the CRISPR-associated (*cas*) genes that code for proteins involved in the CRISPR/Cas defense response. **(B)** The three-stage CRISPR/Cas defense response. Stage 1, adaptation, occurs when the bacterial or archaeal host recognizes a fragment of DNA or RNA from an invader, named the protospacer, and integrates it into the CRISPR array as a new spacer. New spacers are integrated at the leader end of the array. Stage 2, expression, involves the transcription of the *cas* genes into Cas proteins, and the CRISPR array into a precursor CRISPR RNA (pre-crRNA) molecule. The pre-crRNA molecule then gets processed by Cas proteins into smaller, mature CRISPR RNA (crRNA) molecules. In Stage 3, interference, the mature crRNAs associate with one or more Cas proteins to form crRNA-effector complexes, which survey the cell for foreign nucleic acids and subsequently cleave them *via* recognition of the protospacer-adjacent motif (PAM) sequence.

*C*lustered *r*egularly *i*nterspaced *s*hort *p*alindromic *r*epeats (CRISPRs) and *C*RISPR *as*sociated (Cas) proteins, or CRISPR/Cas, is a bacterial and archaeal DNA-based adaptive immune system that defends against bacteriophages, plasmids, and other mobile genetic invaders (Bhaya et al., 2011; Terns and Terns, 2011). The CRISPR/Cas system was first discovered by Japanese scientists in *Escherichia coli* (Ishino et al., 1987), but it has now been found in a large array of prokaryotic species. Among these species, CRISPR/Cas DNA loci exhibit extensive genetic diversity, but they all have a common underlying architecture comprising a CRISPR array composed of direct repeats interspaced with unique sequences called spacers, which are derived from foreign nucleic acids; this array is flanked by associated *cas* genes organized as an operon (Jansen et al., 2002; Bolotin et al., 2005; Pourcel et al., 2005) (**Figure 1A**). These elements of the CRISPR/Cas system work in concert to direct a three-stage defense response against invading phages and plasmids (Barrangou et al., 2007; van der Oost et al., 2009) (**Figure 1B**). Stage 1 (adaptation) occurs when the bacterial or archaeal host recognizes a fragment of DNA or RNA from an invader, named the protospacer (Deveau et al., 2008), and integrates it into the CRISPR array as a new spacer sequence (van der Oost et al., 2009). Protospacer selection is dictated by the presence of highly conserved 2–3 nucleotide regions near the protospacer sequence called ‘protospacer-adjacent motifs’, or PAMs (Marraffini and Sontheimer, 2010). Stage 2 (expression) involves the transcription of the CRISPR array into large RNA transcripts called precursor CRISPR-derived RNAs (pre-crRNAs), which are processed into smaller, mature crRNAs by Cas proteins that are transcribed from the *cas* genes (van der Oost et al., 2009). A mature crRNA contains a unique phage-derived spacer sequence flanked by fragments of its adjacent repeat sequences from the CRISPR array (Wiedenheft et al., 2012). Finally, Stage 3 (interference) takes place when a subsequent attack occurs by the same bacteriophage or plasmid; each crRNA associates with one or more Cas proteins to form a crRNA-protein effector complex, which conducts surveillance of the cell for invading DNA or RNA (Terns and Terns, 2011). The crRNA acts as a guide for the effector complex (guide RNA), directing it to create a double-stranded break (DSB) in the complementary protospacer sequence of the invader *via* recognition of the PAM (Terns and Terns, 2011). The fragmented DNA or RNA can no longer infect the host, thus completing successful defense by the CRISPR/Cas system. This highly adaptable nucleotide-based activity is what makes CRISPR/Cas gene editing technologies, which are designed from CRISPR/Cas systems, so effective.

### How Does CRISPR/Cas9 Gene Editing Technology Work?

CRISPR/Cas9 gene editing technology is based on the Type II CRISPR/Cas system from the human pathogen *Streptococcus pyogenes* and was developed by an internationally collaborative research group headed by Jennifer Doudna and Emmanuelle Charpentier (Jinek et al., 2012). Type II systems are characterized by the multidomain protein Cas9 (Makarova et al., 2011; Makarova et al., 2015), which relies on two RNA molecules to guide it to its DNA target: a trans-activating crRNA (tracrRNA), and the usual crRNA (Deltcheva et al., 2011; Gottesman, 2011; Jinek et al., 2012). The tracrRNA is complementary to the repeat sequences in the corresponding pre-crRNA, and it base-pairs to those sequences facilitating Cas9 to process the pre-crRNA into a smaller mature crRNA molecule (Deltcheva et al., 2011). The tracrRNA, mature crRNA, and Cas9 endonuclease then form an effector complex and the two RNA molecules guide Cas9 to the target protospacer sequence of an invader (Jinek et al., 2012). Cas9 then uses the complementarity between the crRNA and the protospacer as well as the adjacent PAM to create a DSB in the target DNA (Jinek et al., 2012). The Doudna/Charpentier research group developed a single chimeric RNA molecule that combined the tracrRNA and crRNA, and they demonstrated that Cas9 can be programmed to cleave any target DNA by changing only a 20-nucleotide sequence in this single-guide RNA (sgRNA) (Jinek et al., 2012). Their results were immediately significant for the scientific community, allowing the editing of DNA in a broad range of organisms and caused a surge of research in all scientific fields that continues to this day. Additionally, the original chimeric *S. pyogenes* Cas9 technology has served as a model system from which many different CRISPR technologies have evolved.

### CRISPR/Cas9 Exploits Cellular DNA Repair Mechanisms to Edit Genes 

The success of nuclease-based technologies, including CRISPR/Cas9, as methods to edit genes relies on the highly conserved cellular DNA repair mechanisms present in all domains of life. Cas9 generates blunt-end DSBs at target DNA sites, and there are two main mechanisms that are triggered in eukaryotic cells in response to these DSBs: end-joining and homologous recombination, or homology-directed repair (HDR) (Ranjha et al., 2018). Both end-joining and HDR involve complex endogenous systems that can be divided into several sub-pathways that are triggered under different cellular conditions and generate very different repaired DNA products (Ceccaldi et al., 2016; Ranjha et al., 2018). There are two end-joining pathways, non-homologous end-joining (NHEJ) and microhomology-mediated end-joining (MMEJ), and neither require a DNA template for repair (Ranjha et al., 2018). These end-joining mechanisms are highly error prone, often resulting in insertions or deletions (indels) that create knockout mutants when they occur in the reading frame of a gene (Bortesi and Fischer, 2015; Ranjha et al., 2018). Conversely, repairs by the HDR pathways occur only in the presence of a donor DNA template containing regions homologous to the sequences surrounding the DSB induced by Cas9 (Bortesi and Fischer, 2015). The HDR pathway is more precise than the end-joining mechanisms, and thus can be used for highly specific gene modification or gene insertion. Eukaryotic organisms can use both homologous recombination and end-joining mechanisms to repair DNA damage in their cells, but the end-joining pathways are more frequent because they occur regardless of the presence of a donor DNA template and can therefore take place in any stage of the cell cycle (Ranjha et al., 2018). However, end-joining allows for less control in CRISPR/Cas9 gene editing due to the randomness of the mutations it induces (Langner et al., 2018). The less frequent HDR pathway allows for more control, but requires a homologous donor DNA template that, even when present, triggers HDR at a much lower rate than end-joining (Langner et al., 2018). Consequently, researchers wishing to activate the HDR pathway using CRISPR/Cas9 have the additional task of designing a homologous donor template that can be used for recombination at the target DNA site, and they will likely have to screen larger numbers of transformants to identify successful HDR candidates.

### CRISPR/Cas9 Limitations

The major limitation of using the original *S. pyogenes* CRISPR/Cas9 (*Sp*Cas9) is the requirement of the PAM sequence adjacent to the protospacer DNA, which is used by the Cas9 complex in conjunction with the complementary sgRNA region to recognize and cleave the target DNA sequence (Jinek et al., 2012). The *Sp*Cas9 complex recognizes the PAM sequence 5′-NGG-3′, where N represents any of the four nucleotide bases (Jinek et al., 2012). This three-base-pair sequence is a common occurrence in most genomes, but its requirement limits the genes that can be targeted, especially when attempting to study genes involved in highly specific pathways of interest (Langner et al., 2018). Additionally, research has shown that CRISPR/Cas9 can recognize alternative PAM sequences, which increases the likelihood of off-target mutations (Zhang et al., 2014). In response to this limitation researchers have developed three new CRISPR/Cas9 systems from Cas9 orthologs in other bacterial species, each of which recognizes a unique PAM: *Sa*Cas9 from *Staphylococcus aureus*, *St*Cas9 from *Staphylococcus thermophilus*, and *Nm*Cas9 from *Neisseria meningitidis* (Kleinstiver et al., 2015). In addition, Cas9 protein variants are being engineered to recognize alternative PAMs (Agudelo et al., 2020). New CRISPR/Cas nucleases from other bacterial Type II systems have also been discovered: Cas12a, which targets DNA, and Cas13a, which targets single-stranded RNA (Shmakov et al., 2015; Burstein et al., 2017; Koonin et al., 2017). While bearing some similarities to Cas9, these two systems use slightly different mechanisms for cleaving target nucleic acid sequences and processing of pre-crRNA and demonstrate advantages over Cas9 for certain applications, including within plant pathology (Langner et al., 2018).

Another limitation of any CRISPR/Cas technology is the occurrence of unwanted mutations (translocations, inversions, large deletions and insertions) resulting from the complex endogenous pathways that repair the double-stranded DNA breaks induced by Cas nucleases (Després et al., 2018; Kosicki et al., 2018). Additionally, Cas9-induced DSBs can be toxic to cells, inducing cell-death pathways and resulting in low transformation and editing efficiencies (Garst et al., 2017; Roy et al., 2018). To avoid these DSB-related limitations, nuclease-deficient Cas9 proteins have been engineered and fused to other proteins such as deaminases and recombinases to achieve base editing and site-specific recombination (Nishida et al., 2016; Standage-Beier et al., 2019). However, Cas9 continues to be the most commonly used CRISPR/Cas technology, has a number of applications in plant pathology, and is a valuable untapped resource for forest pathology; it is therefore the focus of the remainder of this review.

## Current Applications of CRISPR/Cas9 in Plant Pathology

Plant pathogenic viruses, bacteria, oomycetes, and fungi are natural components of healthy ecosystems, but globalization, climate change, and mismanagement have led many of these species to cause emerging infectious diseases (EIDs) that threaten natural and managed plant ecosystems (Anderson et al., 2004; Fisher et al., 2012). In the context of agriculture, plant EIDs are considered a threat to global food security (Pennisi, 2010), and in forestry they have significant impacts from both economic and biodiversity conservation perspectives (Anderson et al., 2004; Fisher et al., 2012). Chemical mitigation strategies using pesticides and fungicides have proven to be inadequate and environmentally destructive (Andolfo et al., 2016), so research has turned to genetic strategies: developing disease resistance in plants and/or engineering avirulent strains of pathogens. The development of CRISPR/Cas9 as an accurate and versatile gene editing technology increased the scope of such genetic strategies and has led plant pathologists to explore its disease-mitigation applications in both hosts and pathogens.

### Using CRISPR/Cas9 to Engineer Disease Resistance in Plants

To date, most of the CRISPR/Cas9 research in plant pathology has focused on developing systems in the hosts, namely engineering disease resistance in agriculturally important plants. Not surprisingly, the first plants to be engineered using CRISPR/Cas9 were the model species *Arabidopsis thaliana* (Feng et al., 2013) and *Nicotiana benthamiana* (Nekrasov et al., 2013), but these were followed almost simultaneously by development in rice (Feng et al., 2013; Jiang et al., 2013; Miao et al., 2013), wheat (Wang et al., 2014), sorghum (Jiang et al., 2013), maize (Liang et al., 2014), and tomato (Brooks et al., 2014). The list of plant species engineered using CRISPR/Cas9 has expanded rapidly in the last six years, but it has remained in the realm of angiosperm species important in agriculture; use of this technology in forest species is largely absent in the literature, with the one exception of studies developing CRISPR/Cas9 systems in *Populus* species (Fan et al., 2015; Zhou et al., 2015).

The first CRISPR/Cas9 studies in plants were proof-of-concept experiments demonstrating the use of this technology in plants, but many of these species have now been engineered for resistance to viral, fungal, and bacterial diseases (Das et al., 2019). Engineering disease resistance in plants using CRISPR/Cas9 has generally been executed using one of two strategies: the pathogen–gene approach or the plant-gene approach. The former involves engineering an sgRNA into the plant chromosome that directs Cas9 to target a specific pathogen gene thereby impeding pathogenesis, whereas the latter uses an sgRNA that targets endogenous plant genes involved in pathogen interactions and modifies them to either boost the host immune response, or to interfere with the host-recognition pathway of the pathogen (Makarova et al., 2018).

#### Pathogen-Gene Approach

The pathogen-gene approach is best demonstrated with plant-virus pathosystems. CRISPR/Cas9-mediated virus resistance is most commonly tackled *via* a transgenic approach whereby a viral DNA sequence is used to design the sgRNA and is transformed into the plant genome with the CRISPR/Cas9 system (Ali et al., 2015; Ali et al., 2016; Zhang et al., 2018). This induces a response remarkably similar to that of the native CRISPR/Cas systems in that the plant is able to use its transgenic sgRNA-Cas9 system to target invading virus DNA, RNA, or mRNA. This approach has been primarily used in the model species *Nicotiana benthamiana* (Ali et al., 2015; Ali et al., 2016; Ji et al., 2018; Zhang et al., 2018) and *Arabidopsis thaliana* (Ji et al., 2018; Zhang et al., 2018), but it presents an intriguing paradigm for the use of CRISPR/Cas9 to fortify plant immune systems against invading pathogens.

#### Plant-Gene Approach

Engineering CRISPR/Cas9-mediated disease resistance using the plant-gene approach has mostly focused on targeting plant susceptibility (*S*) genes, a diverse group of genes with varying roles that ultimately render plants more susceptible to invading pathogens. The proteins encoded by *S* genes fall into two general categories: those that act as negative regulators of immunity, decreasing the plant immune response in certain contexts, and those that are part of plant development and regulatory pathways, which are targets for pathogen effector molecules (Langner et al., 2018). While traditional disease-resistance breeding has focused on disease resistance (*R*) genes that generally involve ‘gene-for-gene’ interactions with pathogens, it is thought that targeting *S* genes will yield more stable, broad-spectrum disease resistance (Pavan et al., 2009). Both *R*-gene- and *S*-gene-based resistance involve complex molecular pathways that interact with pathogens in different ways, and the details of these interactions have been reviewed elsewhere (Dangl and Jones, 2001; Jones and Dangl, 2006; Pavan et al., 2009; Lapin and Van den Ackerveken, 2013). Engineering resistance to viruses with CRISPR/Cas9 using the plant-gene approach involves designing the sgRNA to target a region of the plant genome that is used by the virus for replication (Makarova et al., 2018). This method has been used for disrupting RNA viruses in both *Cucumis sativus* (cucumber) and *A. thaliana* by targeting the eukaryotic translation initiation factor gene *eIF4E* in the plant (Chandrasekaran et al., 2016; Pyott et al., 2016). However, the plant-gene approach is best demonstrated in pathosystems involving bacteria, oomycetes, and fungi in which the proteins encoded by plant *S* genes are relied upon by these pathogens for host recognition and immune suppression. Generating plant resistance in these systems has predominantly been focused on designing sgRNAs to target *S* genes, creating host knockout mutants that the pathogen effectors have difficulty recognizing (Langner et al., 2018; Das et al., 2019).

A well-studied *S* gene system is the mildew resistance locus O (*MLO*), which renders both monocot and dicot plant hosts susceptible to a variety of powdery mildew pathogens. The use of CRISPR/Cas9 to disrupt the *MLO* gene has proven to be effective for evading pathogen effector recognition and generating disease resistance in these systems. One of the best examples of this is a study by Nekrasov et al. who used CRISPR/Cas9 to generate 48 bp deletions in an *Mlo* gene in tomato plants (Nekrasov et al., 2017). The CRISPR/Cas9 mutants demonstrated resistance to the powdery mildew fungus *Oidium neolycopersici* without generating any other unwanted phenotypic effects (Nekrasov et al., 2017). Second-generation progeny (F1) were then cultivated by selfing the first-generation resistant mutants (F0), which resulted in the CRISPR/Cas9 transfer DNA plasmid being segregated away (Nekrasov et al., 2017). The F1 progeny also exhibited *O. neolycopersici* resistance, and whole-genome sequencing showed that no off-target mutations had occurred, and that no transgenic DNA was present (Nekrasov et al., 2017). A similar study by Wang et al. generated resistance in rice plants (*Oryza sativa*) to the rice blast pathogen *Magnaporthe oryzae* by using CRISPR/Cas9 to induce indels in an *S* gene that encodes proteins involved in sugar transport (SWEET proteins) (Wang et al., 2016). Wang et al. also used segregation to create non-transgenic, disease-resistant F1 progeny that exhibited all the desirable phenotypes from the wild-type plants (Wang et al., 2016). There are now a number of examples of CRISPR/Cas9 *S* gene mutants with enhanced disease resistance to various pathogens including: broad virus resistance in cucumber plants through disruption the eukaryotic translation initiation factor *eIF4E* (Chandrasekaran et al., 2016), resistance to bacteria and oomycete pathogens in tomato plants through deletions in a DMR6 gene (de Toledo Thomazella et al., 2016), bacterial canker-resistant Wanjincheng orange plants *via* mutations in the *CsLOB1* gene promoter (Peng et al., 2017), and powdery mildew resistance in wheat through Cas9-mediated mutations of the *TaEDR1* susceptibility gene (Zhang et al., 2017).

The above studies demonstrate the advantage of using the plant-gene approach in CRISPR/Cas9 research because the disruption of these genes with indels and the subsequent segregation of the transfer DNA results in disease-resistant plants that do not contain any transgenic material. However, the backcrossing required to segregate away the CRISPR/Cas9 plasmid DNA is only feasible in annual plants with short life cycles and is not suitable for perennial crop plants or forest species (Kanchiswamy et al., 2015). Non-transgenic CRISPR/Cas9 mutants can also be generated using a plasmid-free delivery system; this involves designing a pre-assembled enzymatic ribonucleoprotein (RNP) Cas9-sgRNA complex that is transfected directly into plant protoplasts. The Cas9-sgRNA RNP complex can modify the genomic target DNA but is subsequently degraded by the cell – this results in a disease-resistant plant mutant that contains no transgenic DNA (Makarova et al., 2018). These non-transgenic approaches are especially relevant in plant-based industries where the introduction of inter-specific transgenes generates public controversy around genetically modified organisms (GMOs) and initiates prohibitively strict regulations surrounding the use of such genetically modified plants. The ability of CRISPR/Cas9 to generate highly specific disease-resistant mutants that contain no foreign DNA allows for these plants to be used outside of the GMO regulatory framework (Kanchiswamy et al., 2015; Kanchiswamy, 2016; Makarova et al., 2018). It also allows specific genetic modifications to be made in the endogenous genomic context thereby avoiding the random insertion of transgenes from unrelated species and removing the risk of any unintended downstream effects from the presence of foreign DNA (Kim et al., 2014; Kanchiswamy et al., 2015; Kanchiswamy, 2016).

### Using CRISPR/Cas9 to Target and Explore Genes in Filamentous Plant Pathogens

While the use of CRISPR/Cas9 to engineer pathogen resistance in plants is a promising approach to mitigating disease outbreaks, using CRISPR/Cas9 in pathogens is of equal interest both for the generation of avirulent strains and for increasing our understanding of how these species interact with their plant hosts to induce disease. So far, CRISPR/Cas9 research in plant pathogens has been far less prolific than research on plant disease resistance, and it has focused primarily on proof-of-concept experiments in filamentous fungi and oomycetes. The first successful demonstrations of CRISPR/Cas9 technology in filamentous fungi were independently published by four research groups in 2015, who all developed CRISPR/Cas9 systems in filamentous ascomycete species including *Neurospora crassa* (Matsu-ura et al., 2015), *Pyricularia oryzae* (Arazoe et al., 2015), *Trichoderma reesei* (Liu et al., 2015), and multiple *Aspergillus* species (Nødvig et al., 2015). These studies used Cas9 genes that were codon-optimized for filamentous fungi, endogenous promoters for expression of the sgRNA, and common fungal selection markers. More proof of concept studies followed and CRISPR/Cas9 was developed in a number of fungal species including important plant pathogens such as *Ustilago maydis* (corn smut: Schuster et al., 2016), *Fusarium graminearum* (Fusarium head blight of grain: Gardiner and Kazan, 2018), *F. oxysporum* (Fusarium wilt disease: Wang et al., 2018), and *Sclerotinia sclerotiorum* (white mold: Li et al., 2018). The first CRISPR/Cas9 system in oomycetes was developed in the soybean pathogen *Phytophthora sojae*; the study used a Cas9 gene with human-optimized codons fused to the *P. sojae* nuclear localization signal (PsNLS) and driven by the oomycete Ham34 promoter (Fang and Tyler, 2016). CRISPR/Cas9 has now additionally been developed in *P. capsici* (Miao et al., 2018) and *P. palmivora* (Gumtow et al., 2018). A comprehensive review of the CRISPR/Cas9 techniques being used in filamentous fungi and oomycetes has been published by Schuster and Kahmann (2019); as with the plants, the CRISPR/Cas9 studies in filamentous pathogens have been focused on agriculturally important species, with no studies on forest pathogens reported in our literature search.

#### Targeting Pathogenicity Genes Using CRISPR/Cas9

The study on *S. sclerotiorum* by Li et al. (2018) is one of the few that has used CRISPR/Cas9 to generate pathogenicity mutants in a fungal plant pathogen. Their gene target was the oxalate biosynthesis gene *Ssoah1*, responsible for producing oxalic acid, which is involved in host tissue colonization by *S. sclerotiorum*. Li et al. generated DSBs at multiple *Ssoah1* target sites and found that fragments of their Cas9 transformation plasmid had been integrated into the *S. sclerotiorum* genome at the DSB sites; this demonstrated that the transformation plasmid was not only providing the Cas9 protein and sgRNA molecule, but also acting as a donor DNA molecule for the NHEJ pathway to repair the Cas9-induced DSB (Li et al., 2018). The Cas9-generated *S. sclerotiorum* *Ssoah1*-mutant strains exhibited significantly reduced oxalic acid production and reduced pathogenicity on soybean, Abyssinian cabbage, and tomato plants (Li et al., 2018). *Sclerotinia sclerotiorum* is a highly aggressive, necrotrophic phytopathogen with a very broad host range, so these results by Li et al. are very encouraging for the use of CRISPR/Cas9 as a tool for understanding the virulence of similar plant pathogens. Improved understanding of the specific modes of pathogenicity employed by different phytopathogens will subsequently improve management strategies for the diseases they cause.

An important group of pathogenicity genes are those encoding the effector proteins secreted by pathogens during host interactions. Effectors are an extremely diverse group of molecules and are found in some form in all groups of plant pathogens; they have a number of functions including facilitating infection, disrupting the plant immune response, and obtaining nutrients from host tissues (Toruño et al., 2016). Their ubiquity, as well as their dominant role in plant–pathogen interactions, makes them excellent candidates for CRISPR/Cas9 research. This was demonstrated by Fang and Tyler in the first study to develop a CRISPR/Cas9 system in oomycetes; they designed an sgRNA that targeted the RxLR effector gene *Avr4/6* in the soybean pathogen *Phytophthora sojae* (Fang and Tyler, 2016). RxLR effectors are widespread in oomycetes and can enter host cells and suppress effector-triggered immune responses (Jiang and Tyler, 2012). They can be detected by the receptors encoded by plant *R* genes (Jiang and Tyler, 2012); in soybeans the *R* genes involved in recognition of the Avr4/6 effector protein are *Rps*4 and *Rps*6 (Fang and Tyler, 2016). Fang and Tyler examined both the pathogen and host aspects of the *P. sojae* pathosystem: they used CRISPR/Cas9 to create five homozygous NHEJ *Avr4/6* mutants and two homozygous HDR *Avr4/6* mutants, and then assessed the interactions of the *P. sojae* mutants with soybean plants both with and without *Rps*4 or *Rps*6 resistance loci (Fang and Tyler, 2016). Their results showed that when inoculated with *P. sojae*-*Avr4/6* mutants, the *Rps4/Rps6* soybean plants were less able to defend themselves against infection, exhibiting an impaired immune response (Fang and Tyler, 2016).

The results from Fang and Tyler’s 2016 study demonstrate the intricacy of plant–pathogen interactions and serve as a reminder for researchers wishing to use genetic engineering technologies to develop disease-resistant plants: using CRISPR/Cas9 to target an effector may impede the pathogen, but it might also have unintended consequences for the plant host depending on the recognition pathway associated with the effector. Systems that have coevolved for millions of years cannot be easily deconstructed, and if CRISPR/Cas9 is to be used as a tool for mitigating plant disease outbreaks the complexities of these systems must be considered. However, CRISPR/Cas9 provides a perfect opportunity to understand these complex pathosystems, and the study by Fang and Tyler is an excellent example of the use of CRISPR/Cas9 as a tool for elucidating the roles of pathogen genes *via* a functional genomics approach.

## Future Perspectives for CRISPR/Cas9 in Forest Pathology

**Figure 2:**
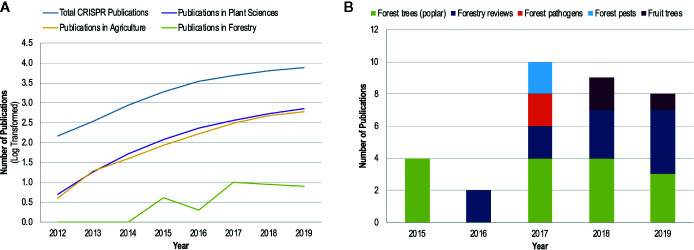
The number of CRISPR publications by year since 2012 (development of CRISPR/Cas9 gene editing technology by Jinek et al.) obtained from a Web of Science topic search with the search parameters: ‘CRISPR’ or ‘CRISPR-Cas*’, or ‘CRISPR/Cas*’. The ‘Analyze Results’ function of Web of Science was used to determine publication numbers by year and research area. **(A)** The log-transformed number of CRISPR publications by research area shows the common trend of increasing publications for CRISPR research for all areas except forestry. **(B)** A breakdown of the publication distribution by topic within forestry as determined by a manual search of the Web of Science search results.

Given the large number of studies on CRISPR/Cas9 in plant pathology and the promises for developing disease resistance, it is somewhat surprising that there is very little literature in the area of forest pathology (**Figure 2**). Forest pathogens are as devastating as their agricultural counterparts and can cause landscape level mortality that results in ecosystem-wide changes; well-known global examples include chestnut blight, Dutch elm disease, ash dieback, myrtle rust, white pine blister rust, and sudden oak death. However, disease management options are more limited in forestry than in agriculture; for example, the use of fungicides is generally confined to forest nurseries, with chemicals rarely being applied to trees once they have been planted. Disease resistance is one of the most promising avenues to combat forest pathogens, especially given the large geographical scales that these pathogens can affect. While there is recognition of the potential applications of CRISPR/Cas9 for forest ecosystems and a call for the use of this technology in these systems (Tsai and Xue, 2015; Fernandez i Marti and Dodd, 2018; Fritsche et al., 2018), no applied studies in forest pathosystems have been performed.

From the perspective of engineering plant disease resistance, this lack of CRISPR/Cas9 research in forest species is understandable for three major reasons. The first is that conifer species, which dominate forest ecosystems in the Northern Hemisphere, have significantly larger and more complex genomes than most agricultural angiosperms. Thus, there are far fewer whole genome sequences available (Nystedt et al., 2013; Neale et al., 2017)—a requirement for CRISPR/Cas9 gene editing in order to design effective sgRNAs and minimize off-target effects. The second reason is the increased difficulty of transformation protocols for forest tree species; this requires not only the transformation of DNA, but also the subsequent regeneration of the whole plant, which is a more time-consuming and complex process in woody perennial plants (Peña and Séguin, 2001; Fernandez i Marti and Dodd, 2018). The final reason is the controversy surrounding GMOs, which is shared with the agricultural sector, but is perhaps greater for woody forest species given their perennial nature and existence within semi-natural ecosystems; furthermore, the regulatory restrictions on genetically engineered trees are far stricter than those for agricultural crops (Strauss et al., 2009; Strauss et al., 2015; Strauss et al., 2016). Given these limitations, very few woody perennials have been successfully engineered with CRISPR/Cas9 relative to their annual counterparts, and the list of those that have is almost exclusively made up of agricultural species including *Coffea canephora* (coffee: Breitler et al., 2018), *Citrus sinensis* (sweet orange: Jia and Wang, 2014), *Citrus paradisi* (Duncan grapefruit: Jia et al., 2016), and *Malus* (apple: Malnoy et al., 2016; Nishitani et al., 2016). The only forest species for which CRISPR/Cas9 systems have been developed are those in the genus *Populus*: *P. tomentosa* (Fan et al., 2015; Jiang et al., 2017; Wan et al., 2017; Wang et al., 2017; Xu et al., 2017; Yang et al., 2017; Shen et al., 2018), *P. tremula* × *alba* (Zhou et al., 2015; Muhr et al., 2018), and *P. tremula* x *tremuloides* (Elorriaga et al., 2018). Only two of these studies used CRISPR/Cas9 to target genes involved in poplar disease resistance (Jiang et al., 2017; Wang et al., 2017).

**Figure 3:**
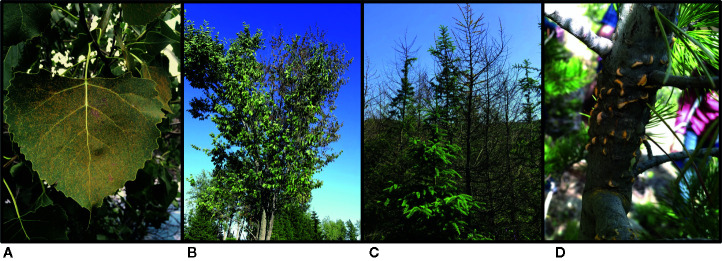
The four forest pathosystems proposed for future CRISPR/Cas9 research: **(A)** uredinia of the poplar leaf rust pathogen *Melampsora medusae* f. sp. *deltoidis*, a basidiomycete fungus, on eastern cottonwood (*Populus deltoides*) leaves in Montréal, Quebec, CA (photo: Richard Hamelin, 2015); **(B)** flagging symptoms typical of Dutch elm disease, caused by the ascomycete fungal pathogen *Ophiostoma novo-ulmi*, on an American elm (*Ulmus americana*) in Québec, Quebec, CA (photo: Philippe Tanguay, 2016); **(C)** sudden larch death caused by the oomycete pathogen *Phytophthora ramorum* in a larch plantation in Galloway Forest Park, Scotland, GB (photo: Richard Hamelin, 2019); **(D)** aecia of the basidiomycete fungal pathogen *Cronartium ribicola*, causal agent of white pine blister rust, on a limber pine (*Pinus flexilis*) in Rocky Mountain National Park, Colorado, US (photo: Erika Dort, 2019).

Given the paucity of studies using CRISPR/Cas9 in forest tree species, it is not surprising that there are no published examples of the use of this technology in forest pathogens. However, there should be fewer obstacles for applying this approach to the pathogens since they are often taxonomically related to the oomycete and fungal species that cause disease on agricultural plants. The study by Fang and Tyler on *Phytophthora sojae* (2016) clearly demonstrated the power of CRISPR/Cas9 as a tool for gaining a deeper understanding of phytopathogens and how they interact with their plant hosts. Similar studies should be implemented in forest pathogens, and there are some forest pathosystems that are ideally suited for the use of CRISPR/Cas9 technology. Below, we give examples of four pathosystems that could be used to drive CRISPR/Cas9 research development in forest pathology (**Figure 3**): two using an approach of engineered resistance in the host, and two using an exploratory approach in the pathogen.

### Proposed CRISPR/Cas9 Research in Four Forest Pathosystems

#### Engineering Disease Resistance in Poplars

While CRISPR/Cas9 research in poplars has already started, its focus has mainly been on modifying genes involved in growth, reproduction, and lignin development with the goal of improving *Populus* species for growth in plantations as well as use in the pulp and bio-refinery industries (Fan et al., 2015; Zhou et al., 2015; Wan et al., 2017; Xu et al., 2017; Yang et al., 2017; Elorriaga et al., 2018; Muhr et al., 2018; Shen et al., 2018; Chanoca et al., 2019). However, there are many potential applications for CRISPR/Cas9 technology for developing disease resistance, and perhaps the most promising application in forest pathology would be the genome editing of poplar species for resistance to their major pathogens, namely *Melampsora* and *Sphaerulina* species. In fact, the *Melampsora*-*Populus* pathosystems (**Figure 3A**) have previously been proposed as a model system to further our understanding of host–pathogen recognition mechanisms and the infection process (Feau et al., 2007) and would therefore be an excellent place to start CRISPR/Cas9 research in forest pathology.

Poplars are ecologically and economically valuable trees, playing important roles in both natural and managed forests, and they have long been established as model species for molecular and genomic studies in forest trees (Bradshaw et al., 2000; Taylor, 2002; Wullschleger et al., 2002). Additionally, *Populus trichocarpa* was the first tree species to have its genome sequenced (Tuskan et al., 2006), which perfectly situates poplar pathosystems for study with CRISPR/Cas9. *Melampsora* leaf rusts and *Sphaerulina* leaf spot and stem canker pathogens are two of the most damaging groups of fungi affecting poplars in both natural stands and plantations, and they have major impacts on productivity and forest health. The barrier of entry for using CRISPR/Cas9 to engineer resistance to these pathogens is particularly low for two reasons, the first being that CRISPR/Cas9 protocols have already been established in *Populus* species (Fan et al., 2015; Zhou et al., 2015). The second reason is that candidate genes associated with resistance have already been identified for both *Melampsora* and *Sphaerulina* species (Yin et al., 2004; Duplessis et al., 2009; La Mantia et al., 2013; Muchero et al., 2018). As discussed previously, there have now been a number of studies using CRISPR/Cas9 in *Populus* species, and while two of these studies used CRISPR/Cas9 to explore the functions of genes in *Melampsora* resistance by creating knockout mutants (Jiang et al., 2017; Wang et al., 2017), none of them used CRISPR/Cas9 to directly engineer disease resistance.

#### Dutch Elm Disease

The possibility of using CRISPR/Cas9 to control the dominant Dutch elm disease (DED) pathogen *Ophiostoma novo-ulmi* (**Figure 3B**) is another exciting prospect. Genome sequencing and annotation of this pathogen has helped in identifying multiple candidate genes involved in the infection process (Forgetta et al., 2013; Khoshraftar et al., 2013; Comeau et al., 2015). Of particular interest are the genes involved in regulating the yeast-mycelial dimorphism exhibited by many *Ophiostoma* species. *Ophiostoma novo-ulmi* uses its budding yeast phase to travel rapidly through the tissues of its elm hosts but can also switch to a mycelial form that can penetrate xylem tissues and grow radially in the elm (Berrocal et al., 2012). This ability to switch between yeast and mycelial growth forms is thought to be involved in the pathogenicity of dimorphic fungi (Nadal et al., 2008), and has thus been explored in the DED pathogens for a number of years (Richards, 1994; Berrocal et al., 2012; Naruzawa and Bernier, 2014; Wedge et al., 2016). Transcriptomic analyses have identified candidate genes involved in this yeast-to-hypha transition (Nigg et al., 2015; Nigg and Bernier, 2016). These genes are excellent candidates for CRISPR/Cas9 gene editing: triggering the NHEJ pathway could create knockout mutants with reduced ability to switch to the yeast form thereby impeding translocation of the fungus throughout the elm tree.

Another group of interest as candidate genes involved in *O. novo-ulmi* pathogenicity are the secondary metabolite clusters. In plant pathogenic fungi, secondary metabolites such as host-selective toxins are well known to play an important role in disease development (Macheleidt et al., 2016). Bioinformatic annotations have identified *O. novo-ulmi* gene clusters putatively involved in biosynthesis of secondary metabolites, and interspecific comparative genomic analyses uncovered a fujikurin-like gene cluster (OpPKS8), found in the DED pathogens (*O. ulmi* and *O. novo-ulmi*) but absent in related non-phytopathogenic species (Sbaraini et al., 2017). According to phylogenetic analyses the authors suggested that this toxin-related cluster may have been horizontally acquired by DED pathogens (Sbaraini et al., 2017). Genes in the OpPKS8 cluster are good candidates for exploration with CRISPR/Cas9, which could be used as an additional tool to elucidate the functions of this secondary metabolite cluster and its potential role in pathogenicity.

Finally, a recent pangenomic analyses of a collection of strains from *O. ulmi* and *O. novo-ulmi* species showed that introgression has been the main driver of genomic diversity and has impacted fitness-related traits, with many of the introgressed regions containing genes involved in host–pathogen interactions and eproduction (Hessenauer et al., 2020). Hessenauer et al. (2020) further demonstrated that the virulence of *O. novo-ulmi* was positively or negatively affected depending on the location of the introgressed genes in the genome. As with the secondary metabolites, CRISPR/Cas9 could be used as a means of exploring the functions of some of these introgressed genes that appear to play a role in virulence. Development of a CRISPR/Cas9 system in *O. novo-ulmi* has already begun (Tanguay, 2019), which makes implementing such pathogenicity-related strategies an impending reality.

#### Sudden Oak/Larch Death 

Some filamentous plant pathogens exhibit virulence *via* their ability to suddenly switch lifestyles. This is the case in many fungal and oomycete species that are hemibiotrophs, meaning they can transition from an asymptomatic biotrophic phase to an aggressive necrotrophic phase in which they begin releasing toxins and killing host tissues (Lee and Rose, 2010; Koeck et al., 2011). The genes enabling this dual lifestyle could be effective targets for CRISPR/Cas9 in order to better understand how hemibiotrophic pathogens cause disease. Research in the oomycete pathogen, *Phytophthora infestans*, showed that effectors are the mediators of this lifestyle transition (Lee and Rose, 2010), which is not surprising given the dominant role these molecules play in plant–pathogen interactions. These results are very encouraging as CRISPR/Cas9 systems have already been developed in *P. sojae* (Fang et al., 2017), *P. capsici* (Miao et al., 2018), and *P. palmivora* (Gumtow et al., 2017), and it has proven successful in disrupting effector genes (Fang and Tyler, 2016; Gumtow et al., 2017). The invasive forest pathogen *Phytophthora ramorum* (**Figure 3C**), causal agent of sudden oak death in the United States (Rizzo et al., 2002a; Rizzo et al., 2002b) and sudden larch death in the United Kingdom (Webber et al., 2010), is another hemibiotrophic oomycete pathogen that causes devastating disease outbreaks in a broad range of woody hosts (Rizzo and Garbelotto, 2003). *Phytophthora ramorum* is classified as a highly aggressive pathogen given its ability to infect woody stems as well as foliar tissues, however, its hemibiotrophic nature allows it to remain asymptomatic anywhere from months to years before it transitions to its aggressive necrotrophic lifestyle (Rizzo and Garbelotto, 2003). It would therefore be an excellent candidate for CRISPR/Cas9 research exploring the role of effectors in mediating this lifestyle switch that influences virulence so significantly.

#### White Pine Blister Rust 

White pine blister rust (WPBR), caused by the basidiomycete rust fungus *Cronartium ribicola* (**Figure 3D**), has severely affected North American populations of many economically and ecologically important pine species such as eastern and western white pines, sugar pine, and whitebark pine (Sniezko et al., 2014). In many white pine species both complete and partial resistance to WPBR have been detected. Complete resistance is mediated by a dominant *R* gene named *Cr*, which causes a hypersensitive response to *C. ribicola* and enables the pine host to survive by restricting the infection to the needles (Kinloch et al., 2003; Sniezko et al., 2014). Partial resistance appears to be a more complex response that is likely mediated by multiple genes, but the exact mechanisms driving this response are not yet known (Sniezko et al., 2014). While the *Cr* genes mediating complete resistance in white pines seem like the perfect candidates for CRISPR/Cas9-generated disease resistance, these *R* genes are likely not stable in the long-term because only a single mutation in a corresponding *C. ribicola* effector gene would be required to overcome this resistance (Sniezko et al., 2014). However, plant *R* gene immune receptors can be mutated to provide resistance to phylogenetically divergent pathogens (Segretin et al., 2014; Giannakopoulou et al., 2015), and CRISPR/Cas9 could be used to engineer such synthetic genes in tandem in order to create stable multi-resistance plant systems (Andolfo et al., 2016). Another option to obtain more durable long-term resistance in WPBR pathosystems is to focus CRISPR/Cas9 research on partial resistance. Cas9 can be co-expressed with many sgRNAs to simultaneously target multiple genes; this multiplex gene editing could facilitate the discovery of the genes involved in partial resistance, and it could also eventually be used to target those genes simultaneously in engineered WPBR-resistant pine populations. This partial resistance strategy may be less effective than complete resistance to a single *C. ribicola* strain, but it would be more stable in the long-term against a constantly evolving *C. ribicola* population exhibiting diverse mutations and could also protect pine hosts against other encroaching pathogen species. 

**Figure 4:**
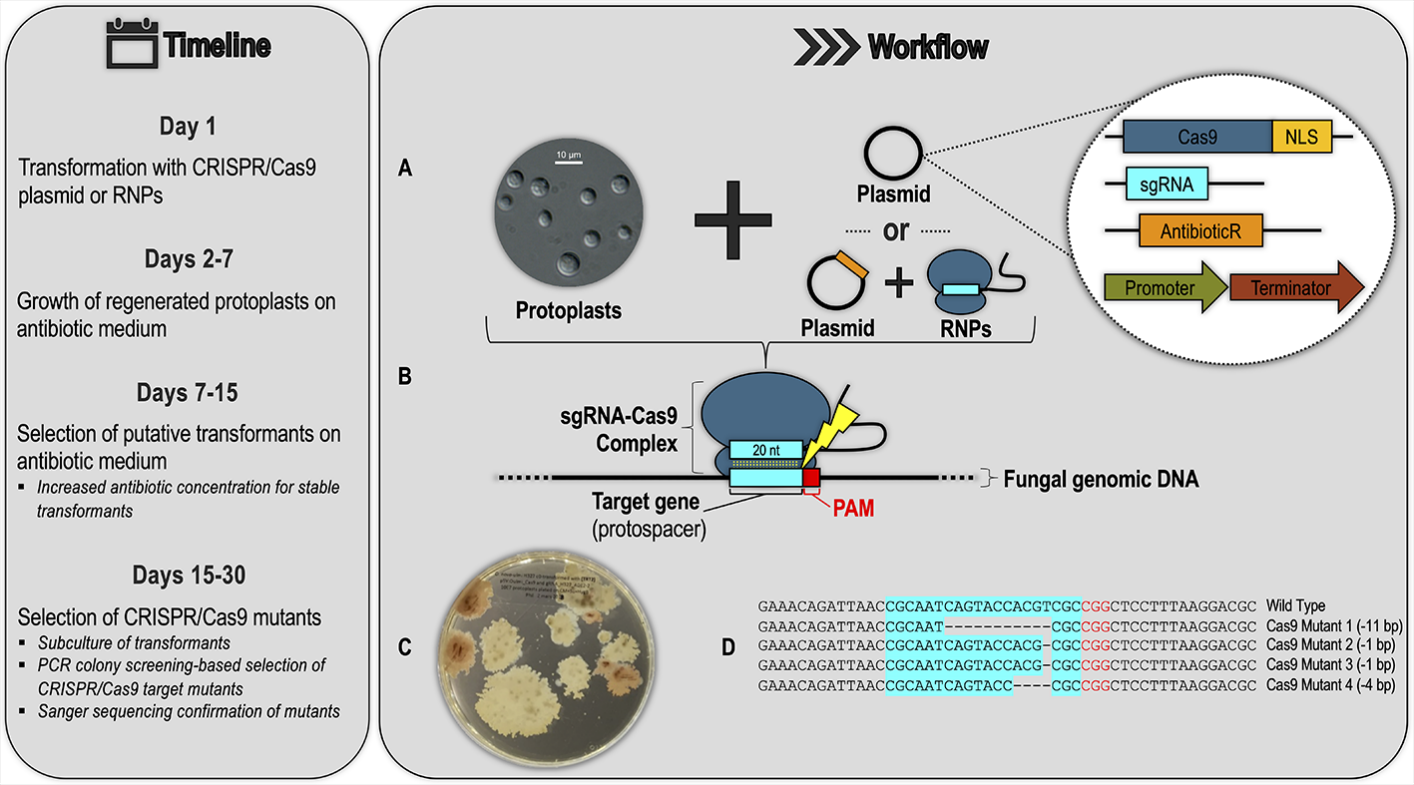
Example of a generalized CRISPR/Cas9 workflow and timeline for a filamentous forest pathogen based on the protocol being developed for the Dutch elm disease pathogen *Ophiostoma novo-ulmi* (*Onu*). **(A)** Transformation of fungal protoplasts with a plasmid containing (see inset): a Cas9 gene fused to nuclear localization signal (NLS), a single-guide RNA (sgRNA) scaffold with a 20-nucleotide (nt) region designed to base-pair with the target genomic DNA, an antibiotic resistance gene for transformant selection, and gene promoters and terminators active in the target species. Alternatively, protoplasts can be co-transformed with a combination of Cas9-sgRNA ribonucleoprotein complexes (RNPs) and a plasmid for antibiotic transformant selection. **(B)** In successful transformants, Cas9 forms a complex with the sgRNA molecule, which guides the complex to the protospacer (a genomic DNA target in this example) with an adjacent PAM (protospacer adjacent motif) sequence. The sgRNA–Cas9 complex is analogous to the crRNA-effector complexes of native CRISPR/Cas systems, shown in **Figure 1** of this review. In the RNP approach, this sgRNA–Cas9 complex is pre-assembled and transfected directly into the protoplasts. **(C)** Transformants are selected by growth on antibiotic selective medium (picture shows *Onu* hygromycin-resistant transformants: pink colonies are CRISPR/Cas9 *ade2* mutants). **(D)** Successful CRISPR/Cas9 mutants are confirmed through sub-culture of the putative transformants from the previous step, PCR screening, and Sanger sequencing.

The four suggestions above demonstrate the scope of utility of CRISPR/Cas9 gene editing technology and highlight how this tool has been underutilized in forest pathology. CRISPR/Cas9 is a relatively recent development, and there are clear obstacles to its use in forest pathosystems. However, given the large number of fungal and oomycete species for which CRISPR/Cas9 systems have now been established, the barrier of entry for pathologists studying filamentous forest pathogens has been lowered, and there is a generalized research pipeline that they can follow to implement this gene editing technology in their study organisms (**Figure 4**). This pipeline can comprise various approaches for developing a CRISPR/Cas9 system, including using different Cas9 delivery methods (RNP vs. plasmid) and genetic targets (DNA vs. RNA). **Figure 4** shows a generalized schematic of one such approach based on the CRISPR/Cas9 system being developed in the Dutch elm disease pathogen *Ophiostoma novo-ulmi* (Tanguay, 2019). We hope this gives forest pathologists a better understanding of the logistics involved in developing CRISPR/Cas9 systems in filamentous forest pathogens.

As sequencing technologies continue to improve and lower in cost, forest pathologists should aim to increase their exploration of the genetic basis of plant disease resistance *via* CRISPR/Cas9 gene editing. The power of this technology to aid our understanding of the intricacies of plant–pathogen interactions and generate effective strategies of disease resistance in forest pathosystems is much too great to ignore.

## Conclusions

Since the discovery of CRISPR/Cas systems in 1987 (Ishino et al., 1987), our understanding of this adaptable immune response has come a long way, and the development of CRISPR/Cas9 gene editing technology in 2012 (Jinek et al., 2012) resulted in an explosion of research with wide-reaching implications for most biological systems. Despite the development of Cas9 tools in many pathosystems, there are still limitations to the use of CRISPR/Cas technology in plant pathology, especially concerning off-target effects. However, careful design of sgRNAs and modifications of the Cas proteins prevent most of these effects, and the use of RNP delivery systems has reduced off-target mutations to zero in many systems (Das et al., 2019). Additionally, the continued development of CRISPR/Cas technology in plant pathosystems will only improve efficiency as this technology is adapted to function in a diversity of organisms. To date, most CRISPR/Cas9 research in plant pathology has been focused on agricultural pathosystems, with little to no research in forest pathology. This is understandable given the availability of genomic resources for most major crops as well as the shorter generation times of crop plants relative to their forest counterparts, which makes for a quick feedback loop on any genetic modifications made with CRISPR/Cas9. However, forest pathogens are wreaking equal havoc in the forestry sector and represent a global threat to forest ecosystems that needs to be addressed immediately. The time is now to adopt CRISPR/Cas9 in forest pathology, at the very least to improve our understanding of host–pathogen interactions, but ideally to begin integrating it into forest improvement programs to generate more effective disease resistance strategies for long-term forest sustainability.

## Author Contributions

All authors listed have made a substantial, direct and intellectual contribution to the work, and approved it for publication.

## Funding

This work was supported by Genome Canada’s Large-Scale Applied Research Project (LSARP project #10106) with additional funding from Genome B.C., Genome Quebec, Canadian Food Inspection Agency, Natural Resources Canada and FPInnovations.

## Conflict of Interest

The authors declare that the research was conducted in the absence of any commercial or financial relationships that could be construed as a potential conflict of interest.

## References

[B1] Agudelo D. Carter S. Velimirovic M. Duringer A. Rivest J.-F. Levesque S. (2020). Versatile and robust genome editing with *Streptococcus thermophilus* CRISPR1-Cas9. Genome Res. 30, 107–117. 10.1101/gr.255414.119 31900288 PMC6961573

[B2] Ali Z. Abulfaraj A. Idris A. Ali S. Tashkandi M. Mahfouz M. M. (2015). CRISPR/Cas9-mediated viral interference in plants. Genome Biol. 16, 238. 10.1186/s13059-015-0799-6 26556628 PMC4641396

[B3] Ali Z. Ali S. Tashkandi M. Zaidi S. S.-A. Mahfouz M. M. (2016). CRISPR/Cas9-Mediated Immunity to Geminiviruses: Differential Interference and Evasion. Sci. Rep. 6, 26912. 10.1038/srep26912 27225592 PMC4881029

[B4] Anderson P. K. Cunningham A. A. Patel N. G. Morales F. J. Epstein P. R. Daszak P. (2004). Emerging infectious diseases of plants: pathogen pollution, climate change and agrotechnology drivers. Trends Ecol. Evol. 19, 535–544. 10.1016/j.tree.2004.07.021 16701319

[B5] Andolfo G. Iovieno P. Frusciante L. Ercolano M. R. (2016). Genome-Editing Technologies for Enhancing Plant Disease Resistance. Front. Plant Sci. 7, 1813. 10.3389/fpls.2016.01813 27990151 PMC5130979

[B6] Arazoe T. Miyoshi K. Yamato T. Ogawa T. Ohsato S. Arie T. (2015). Tailor-made CRISPR/Cas system for highly efficient targeted gene replacement in the rice blast fungus. Biotechnol. Bioeng. 112, 2543–2549. 10.1002/bit.25662 26039904

[B7] Barrangou R. Fremaux C. Deveau H. Richards M. Boyaval P. Moineau S. (2007). CRISPR Provides Acquired Resistance Against Viruses in Prokaryotes. Science 315, 1709–1712. 10.1126/science.1138140 17379808

[B8] Berrocal A. Navarrete J. Oviedo C. Nickerson K. W. (2012). Quorum sensing activity in *Ophiostoma ulmi*: effects of fusel oils and branched chain amino acids on yeast-mycelial dimorphism. J. Appl. Microbiol. 113, 126–134. 10.1111/j.1365-2672.2012.05317.x 22519968

[B9] Bhaya D. Davison M. Barrangou R. (2011). CRISPR-Cas Systems in Bacteria and Archaea: Versatile Small RNAs for Adaptive Defense and Regulation. Annu. Rev. Genet. 45, 273–297. 10.1146/annurev-genet-110410-132430 22060043

[B10] Bolotin A. Quinquis B. Sorokin A. Ehrlich S. D. (2005). Clustered regularly interspaced short palindrome repeats (CRISPRs) have spacers of extrachromosomal origin. Microbiology 151, 2551–2561. 10.1099/mic.0.28048-0 16079334

[B11] Bortesi L. Fischer R. (2015). The CRISPR/Cas9 system for plant genome editing and beyond. Biotechnol. Adv. 33, 41–52. 10.1016/j.biotechadv.2014.12.006 25536441

[B12] Bradshaw H. D. Ceulemans R. Davis J. Stettler R. (2000). Emerging Model Systems in Plant Biology: Poplar (*Populus*) as A Model Forest Tree. J. Plant Growth Regul. 19, 306–313. 10.1007/s003440000030

[B13] Breitler J.-C. Dechamp E. Campa C. Zebral Rodrigues L. A. Guyot R. Marraccini P. (2018). CRISPR/Cas9-mediated efficient targeted mutagenesis has the potential to accelerate the domestication of *Coffea canephora* . Plant Cell Tiss. Organ Cult. 134, 383–394. 10.1007/s11240-018-1429-2

[B14] Brooks C. Nekrasov V. Lippman Z. B. Van Eck J. (2014). Efficient Gene Editing in Tomato in the First Generation Using the Clustered Regularly Interspaced Short Palindromic Repeats/CRISPR-Associated9 System. Plant Physiol. 166, 1292–1297. 10.1104/pp.114.247577 25225186 PMC4226363

[B15] Burstein D. Harrington L. B. Strutt S. C. Probst A. J. Anantharaman K. Thomas B. C. (2017). New CRISPR–Cas systems from uncultivated microbes. Nature 542, 237–241. 10.1038/nature21059 28005056 PMC5300952

[B16] Ceccaldi R. Rondinelli B. D’Andrea A. D. (2016). Repair Pathway Choices and Consequences at the Double-Strand Break. Trends Cell Biol. 26, 52–64. 10.1016/j.tcb.2015.07.009 26437586 PMC4862604

[B17] Chandrasekaran J. Brumin M. Wolf D. Leibman D. Klap C. Pearlsman M. (2016). Development of broad virus resistance in non-transgenic cucumber using CRISPR/Cas9 technology. Mol. Plant Pathol. 17, 1140–1153. 10.1111/mpp.12375 26808139 PMC6638350

[B18] Chanoca A. de Vries L. Boerjan W. (2019). Lignin Engineering in Forest Trees. Front. Plant Sci. 10, 912. 10.3389/fpls.2019.00912 31404271 PMC6671871

[B19] Clarivate Analytics (2020). Results Analysis Tool for Topic: “CRISPR.” Web Sci.

[B20] Comeau A. M. Dufour J. Bouvet G. F. Jacobi V. Nigg M. Henrissat B. (2015). Functional Annotation of the *Ophiostoma novo-ulmi* Genome: Insights into the Phytopathogenicity of the Fungal Agent of Dutch Elm Disease. Genome Biol. Evol. 7, 410–430. 10.1093/gbe/evu281 PMC4350166 25539722

[B21] Dangl J. L. Jones J. D. G. (2001). Plant pathogens and integrated defence responses to infection. Nature 411, 826–833. 10.1038/35081161 11459065

[B22] Das A. Sharma N. Prasad M. (2019). CRISPR/Cas9: A Novel Weapon in the Arsenal to Combat Plant Diseases. Front. Plant Sci. 9, 2008. 10.3389/fpls.2018.02008 30697226 PMC6341021

[B23] de Toledo Thomazella D. P. Brail Q. Dahlbeck D. Staskawicz B. (2016). CRISPR-Cas9 mediated mutagenesis of a *DMR6* ortholog in tomato confers broad-spectrum disease resistance. bioRxiv. 10.1101/064824 PMC8271637 34215692

[B24] Deltcheva E. Chylinski K. Sharma C. M. Gonzales K. Chao Y. Pirzada Z. A. (2011). CRISPR RNA maturation by *trans*-encoded small RNA and host factor RNase III. Nature 471, 602–607. 10.1038/nature09886 21455174 PMC3070239

[B25] Després P. C. Dubé A. K. Nielly-Thibault L. Yachie N. Landry C. R. (2018). Double Selection Enhances the Efficiency of Target-AID and Cas9-Based Genome Editing in Yeast. G3: Genes Genomes Genet. 8, 3163–3171. 10.1534/g3.118.200461 PMC6169390 30097473

[B26] Deveau H. Barrangou R. Garneau J. E. Labonté J. Fremaux C. Boyaval P. (2008). Phage Response to CRISPR-Encoded Resistance in *Streptococcus thermophilus* . J. Bacteriol. 190, 1390–1400. 10.1128/JB.01412-07 18065545 PMC2238228

[B27] Duplessis S. Major I. Martin F. Séguin A. (2009). Poplar and Pathogen Interactions: Insights from *Populus* Genome-Wide Analyses of Resistance and Defense Gene Families and Gene Expression Profiling. Crit. Rev. Plant Sci. 28, 309–334. 10.1080/07352680903241063

[B28] Elorriaga E. Klocko A. L. Ma C. Strauss S. H. (2018). Variation in Mutation Spectra Among CRISPR/Cas9 Mutagenized Poplars. Front. Plant Sci. 9, 594. 10.3389/fpls.2018.00594 29868058 PMC5949366

[B29] Fan D. Liu T. Li C. Jiao B. Li S. Hou Y. (2015). Efficient CRISPR/Cas9-mediated Targeted Mutagenesis in Populus in the First Generation. Sci. Rep. 5, 12217. 10.1038/srep12217 26193631 PMC4507398

[B30] Fang Y. Tyler B. M. (2016). Efficient disruption and replacement of an effector gene in the oomycete *Phytophthora sojae* using CRISPR/Cas9. Mol. Plant Pathol. 17, 127–139. 10.1111/mpp.12318 26507366 PMC6638440

[B31] Fang Y. Cui L. Gu B. Arredondo F. Tyler B. M. (2017). Efficient Genome Editing in the Oomycete *Phytophthora sojae* Using CRISPR/Cas9. Curr. Protoc. Microbiol. 44, 21A.1.1–21A.1.26. 10.1002/cpmc.25 28166383

[B32] Feau N. Joly D. L. Hamelin R. C. (2007). Poplar leaf rusts: model pathogens for a model tree. Can. J. Bot. 85, 1127–1135. 10.1139/B07-102

[B33] Feng Z. Zhang B. Ding W. Liu X. Yang D.-L. Wei P. (2013). Efficient genome editing in plants using a CRISPR/Cas system. Cell Res. 23, 1229–1232. 10.1038/cr.2013.114 23958582 PMC3790235

[B34] Fernandez i Marti A. Dodd R. S. (2018). Using CRISPR as a Gene Editing Tool for Validating Adaptive Gene Function in Tree Landscape Genomics. Front. Ecol. Evol. 6, 76. 10.3389/fevo.2018.00076

[B35] Fisher M. C. Henk D. A. Briggs C. J. Brownstein J. S. Madoff L. C. McCraw S. L. (2012). Emerging fungal threats to animal, plant and ecosystem health. Nature 484, 186–194. 10.1038/nature10947 22498624 PMC3821985

[B36] Forgetta V. Leveque G. Dias J. Grove D. Lyons R. Genik S. (2013). Sequencing of the Dutch Elm Disease Fungus Genome Using the Roche/454 GS-FLX Titanium System in a Comparison of Multiple Genomics Core Facilities. J. Biomol. Tech. 24, 39–49. 10.7171/jbt.12-2401-005 23542132 PMC3526337

[B37] Fritsche S. Poovaiah C. MacRae E. Thorlby G. (2018). A New Zealand Perspective on the Application and Regulation of Gene Editing. Front. Plant Sci. 9, 1323. 10.3389/fpls.2018.01323 30258454 PMC6144285

[B38] Gardiner D. M. Kazan K. (2018). Selection is required for efficient Cas9-mediated genome editing in *Fusarium graminearum* . Fungal Biol. 122, 131–137. 10.1016/j.funbio.2017.11.006 29458716

[B39] Garst A. D. Bassalo M. C. Pines G. Lynch S. A. Halweg-Edwards A. L. Liu R. (2017). Genome-wide mapping of mutations at single-nucleotide resolution for protein, metabolic and genome engineering. Nat. Biotechnol. 35, 48–55. 10.1038/nbt.3718 27941803

[B40] Giannakopoulou A. Steele J. F. C. Segretin M. E. Bozkurt T. O. Zhou J. Robatzek S. (2015). Tomato I2 Immune Receptor Can Be Engineered to Confer Partial Resistance to the Oomycete *Phytophthora infestans* in Addition to the Fungus *Fusarium oxysporum* . MPMI 28, 1316–1329. 10.1094/MPMI-07-15-0147-R 26367241

[B41] Gottesman S. (2011). Dicing defence in bacteria. Nature 471, 588–589. 10.1038/471588a 21455171

[B42] Gumtow R. Wu D. Uchida J. Tian M. (2018). A *Phytophthora palmivora* Extracellular Cystatin-Like Protease Inhibitor Targets Papain to Contribute to Virulence on Papaya. MPMI 31, 363–373. 10.1094/MPMI-06-17-0131-FI 29068239

[B43] Hessenauer P. Fijarczyk A. Martin H. Prunier J. Charron G. Chapuis J. (2020). Hybridization and introgression drive genome evolution of Dutch elm disease pathogens. Nat. Ecol. Evol. 4, 626–638. 10.1038/s41559-020-1133-6 32123324

[B44] Ishino Y. Shinagawa H. Makino K. Amemura M. Nakata A. (1987). Nucleotide sequence of the *iap* gene, responsible for alkaline phosphatase isozyme conversion in *Escherichia coli*, and identification of the gene product. J. Bacteriol. 169, 5429–5433. 10.1128/jb.169.12.5429-5433.1987 3316184 PMC213968

[B45] Jansen R. van Embden J. D. A. Gaastra W. Schouls L. M. (2002). Identification of genes that are associated with DNA repeats in prokaryotes. Mol. Microbiol. 43, 1565–1575. 10.1046/j.1365-2958.2002.02839.x 11952905

[B46] Ji X. Si X. Zhang Y. Zhang H. Zhang F. Gao C. (2018). Conferring DNA virus resistance with high specificity in plants using virus-inducible genome-editing system. Genome Biol. 19, 197. 10.1186/s13059-018-1580-4 30442181 PMC6238286

[B47] Jia H. Wang N. (2014). Targeted Genome Editing of Sweet Orange Using Cas9/sgRNA. PLoS One 9, e93806. 10.1371/journal.pone.0093806 24710347 PMC3977896

[B48] Jia H. Orbovic V. Jones J. B. Wang N. (2016). Modification of the PthA4 effector binding elements in Type I CsLOB1 promoter using Cas9/sgRNA to produce transgenic Duncan grapefruit alleviating XccΔpthA4:dCsLOB1.3 infection. Plant Biotechnol. J. 14, 1291–1301. 10.1111/pbi.12495 27071672 PMC11389130

[B49] Jiang R. H. Y. Tyler B. M. (2012). Mechanisms and Evolution of Virulence in Oomycetes. Annu. Rev. Phytopathol. 50, 295–318. 10.1146/annurev-phyto-081211-172912 22920560

[B50] Jiang W. Zhou H. Bi H. Fromm M. Yang B. Weeks D. P. (2013). Demonstration of CRISPR/Cas9/sgRNA-mediated targeted gene modification in Arabidopsis, tobacco, sorghum and rice. Nucleic Acids Res. 41, e188. 10.1093/nar/gkt780 23999092 PMC3814374

[B51] Jiang Y. Guo L. Ma X. Zhao X. Jiao B. Li C. (2017). The WRKY transcription factors PtrWRKY18 and PtrWRKY35 promote *Melampsora* resistance in *Populus* . Tree Physiol. 37, 665–675. 10.1093/treephys/tpx008 28338710

[B52] Jinek M. Chylinski K. Fonfara I. Hauer M. Doudna J. A. Charpentier E. (2012). A Programmable Dual-RNA–Guided DNA Endonuclease in Adaptive Bacterial Immunity. Science 337, 816–821. 10.1126/science.1225829 22745249 PMC6286148

[B53] Jones J. D. G. Dangl J. L. (2006). The plant immune system. Nature 444, 323–329. 10.1038/nature05286 17108957

[B54] Kanchiswamy C. N. Malnoy M. Velasco R. Kim J.-S. Viola R. (2015). Non-GMO genetically edited crop plants. Trends Biotechnol. 33, 489–491. 10.1016/j.tibtech.2015.04.002 25978870

[B55] Kanchiswamy C. N. (2016). DNA-free genome editing methods for targeted crop improvement. Plant Cell Rep. 35, 1469–1474. 10.1007/s00299-016-1982-2 27100964

[B56] Khoshraftar S. Hung S. Khan S. Gong Y. Tyagi V. Parkinson J. (2013). Sequencing and annotation of the *Ophiostoma ulmi* genome. BMC Genomics 14:162. 10.1186/1471-2164-14-162 23496816 PMC3618308

[B57] Kim S. Kim D. Cho S. W. Kim J. Kim J.-S. (2014). Highly efficient RNA-guided genome editing in human cells via delivery of purified Cas9 ribonucleoproteins. Genome Res. 24, 1012–1019. 10.1101/gr.171322.113 24696461 PMC4032847

[B58] Kinloch B. B. Sniezko R. A. Dupper G. E. (2003). Origin and Distribution of Cr2, a Gene for Resistance to White Pine Blister Rust in Natural Populations of Western White Pine. Phytopathology 93, 691–694. 10.1094/PHYTO.2003.93.6.691 18943055

[B59] Kleinstiver B. P. Prew M. S. Tsai S. Q. Topkar V. V. Nguyen N. T. Zheng Z. (2015). Engineered CRISPR-Cas9 nucleases with altered PAM specificities. Nature 523, 481–485. 10.1038/nature14592 26098369 PMC4540238

[B60] Koeck M. Hardham A. R. Dodds P. N. (2011). The role of effectors of biotrophic and hemibiotrophic fungi in infection. Cell. Microbiol. 13, 1849–1857. 10.1111/j.1462-5822.2011.01665.x 21848815 PMC3218205

[B61] Koonin E. V. Makarova K. S. Zhang F. (2017). Diversity, classification and evolution of CRISPR-Cas systems. Curr. Opin. Microbiol. 37, 67–78. 10.1016/j.mib.2017.05.008 28605718 PMC5776717

[B62] Kosicki M. Tomberg K. Bradley A. (2018). Repair of double-strand breaks induced by CRISPR–Cas9 leads to large deletions and complex rearrangements. Nat. Biotechnol. 36, 765–771. 10.1038/nbt.4192 30010673 PMC6390938

[B63] La Mantia J. Klápště J. El-Kassaby Y. A. Azam S. Guy R. D. Douglas C. J. (2013). Association Analysis Identifies Melampsora ×columbiana Poplar Leaf Rust Resistance SNPs. PLoS One 8, e78423. 10.1371/journal.pone.0078423 24236018 PMC3827267

[B64] Langner T. Kamoun S. Belhaj K. (2018). CRISPR Crops: Plant Genome Editing Toward Disease Resistance. Annu. Rev. Phytopathol. 56, 479–512. 10.1146/annurev-phyto-080417-050158 29975607

[B65] Lapin D. Van den Ackerveken G. (2013). Susceptibility to plant disease: more than a failure of host immunity. Trends Plant Sci. 18, 546–554. 10.1016/j.tplants.2013.05.005 23790254

[B66] Lee S.-J. Rose J. K. C. (2010). Mediation of the transition from biotrophy to necrotrophy in hemibiotrophic plant pathogens by secreted effector proteins. Plant Signaling Behav. 5, 769–772. 10.4161/psb.5.6.11778 PMC3001586 20400849

[B67] Li J. Zhang Y. Zhang Y. Yu P.-L. Pan H. Rollins J. A. (2018). Introduction of Large Sequence Inserts by CRISPR-Cas9 To Create Pathogenicity Mutants in the Multinucleate Filamentous Pathogen *Sclerotinia sclerotiorum* . mBio 9, e00567–e00518. 10.1128/mBio.00567-18 29946044 PMC6020291

[B68] Liang Z. Zhang K. Chen K. Gao C. (2014). Targeted Mutagenesis in *Zea mays* Using TALENs and the CRISPR/Cas System. J. Genet. Genomics 41, 63–68. 10.1016/j.jgg.2013.12.001 24576457

[B69] Liu R. Chen L. Jiang Y. Zhou Z. Zou G. (2015). Efficient genome editing in filamentous fungus *Trichoderma reesei* using the CRISPR/Cas9 system. Cell Discovery 1, 15007. 10.1038/celldisc.2015.7 27462408 PMC4860831

[B70] Macheleidt J. Mattern D. J. Fischer J. Netzker T. Weber J. Schroeckh V. (2016). Regulation and Role of Fungal Secondary Metabolites. Annu. Rev. Genet. 50, 371–392. 10.1146/annurev-genet-120215-035203 27732794

[B71] Makarova K. S. Haft D. H. Barrangou R. Brouns S. J. J. Charpentier E. Horvath P. (2011). Evolution and classification of the CRISPR–Cas systems. Nat. Rev. Microbiol. 9, 467–477. 10.1038/nrmicro2577 21552286 PMC3380444

[B72] Makarova K. S. Wolf Y. II Alkhnbashi O. S. Costa F. Shah S. A. Saunders S. J. (2015). An updated evolutionary classification of CRISPR–Cas systems. Nat. Rev. Microbiol. 13, 722–736. 10.1038/nrmicro3569 26411297 PMC5426118

[B73] Makarova S. S. Khromov A. V. Spechenkova N. A. Taliansky M. E. Kalinina N. O. (2018). Application of the CRISPR/Cas System for Generation of Pathogen-Resistant Plants. Biochem. Moscow 83, 1552–1562. 10.1134/S0006297918120131 30878030

[B74] Malnoy M. Viola R. Jung M.-H. Koo O.-J. Kim S. Kim J.-S. (2016). DNA-Free Genetically Edited Grapevine and Apple Protoplast Using CRISPR/Cas9 Ribonucleoproteins. Front. Plant Sci. 7, 1904. 10.3389/fpls.2016.01904 28066464 PMC5170842

[B75] Marraffini L. A. Sontheimer E. J. (2010). CRISPR interference: RNA-directed adaptive immunity in bacteria and archaea. Nat. Rev. Genet. 11, 181–190. 10.1038/nrg2749 20125085 PMC2928866

[B76] Matsu-ura T. Baek M. Kwon J. Hong C. (2015). Efficient gene editing in Neurospora crassa with CRISPR technology. Fungal Biol. Biotechnol. 2, 4. 10.1186/s40694-015-0015-1 28955455 PMC5611662

[B77] Miao J. Guo D. Zhang J. Huang Q. Qin G. Zhang X. (2013). Targeted mutagenesis in rice using CRISPR-Cas system. Cell Res. 23, 1233–1236. 10.1038/cr.2013.123 23999856 PMC3790239

[B78] Miao J. Chi Y. Lin D. Tyler B. M. Liu X. (2018). Mutations in ORP1 Conferring Oxathiapiprolin Resistance Confirmed by Genome Editing using CRISPR/Cas9 in *Phytophthora capsici* and *P. sojae* . Phytopathology 108, 1412–1419. 10.1094/PHYTO-01-18-0010-R 29979095

[B79] Muchero W. Sondreli K. L. Chen J.-G. Urbanowicz B. R. Zhang J. Singan V. (2018). Association mapping, transcriptomics, and transient expression identify candidate genes mediating plant–pathogen interactions in a tree. PNAS 115, 11573–11578. 10.1073/pnas.1804428115 30337484 PMC6233113

[B80] Muhr M. Paulat M. Awwanah M. Brinkkötter M. Teichmann T. (2018). CRISPR/Cas9-mediated knockout of *Populus* BRANCHED1 and BRANCHED2 orthologs reveals a major function in bud outgrowth control. Tree Physiol. 38, 1588–1597. 10.1093/treephys/tpy088 30265349

[B81] Muñoz I. V. Sarrocco S. Malfatti L. Baroncelli R. Vannacci G. (2019). CRISPR-Cas for Fungal Genome Editing: A New Tool for the Management of Plant Diseases. Front. Plant Sci. 10, 135. 10.3389/fpls.2019.00135 30828340 PMC6384228

[B82] Nadal M. García-Pedrajas M. D. Gold S. E. (2008). Dimorphism in fungal plant pathogens. FEMS Microbiol. Lett. 284, 127–134. 10.1111/j.1574-6968.2008.01173.x 18479435

[B83] Naruzawa E. S. Bernier L. (2014). Control of yeast-mycelium dimorphism in vitro in Dutch elm disease fungi by manipulation of specific external stimuli. Fungal Biol. 118, 872–884. 10.1016/j.funbio.2014.07.006 25442291

[B84] Neale D. B. McGuire P. E. Wheeler N. C. Stevens K. A. Crepeau M. W. Cardeno C. (2017). The Douglas-Fir Genome Sequence Reveals Specialization of the Photosynthetic Apparatus in Pinaceae. G3: Genes Genomes Genet. 7, 3157–3167. 10.1534/g3.117.300078 PMC5592940 28751502

[B85] Nekrasov V. Staskawicz B. Weigel D. Jones J. D. G. Kamoun S. (2013). Targeted mutagenesis in the model plant *Nicotiana benthamiana* using Cas9 RNA-guided endonuclease. Nat. Biotechnol. 31, 691–693. 10.1038/nbt.2655 23929340

[B86] Nekrasov V. Wang C. Win J. Lanz C. Weigel D. Kamoun S. (2017). Rapid generation of a transgene-free powdery mildew resistant tomato by genome deletion. Sci. Rep. 7, 482. 10.1038/s41598-017-00578-x 28352080 PMC5428673

[B87] Nigg M. Bernier L. (2016). From yeast to hypha: defining transcriptomic signatures of the morphological switch in the dimorphic fungal pathogen *Ophiostoma novo-ulmi* . BMC Genomics 17, 920. 10.1186/s12864-016-3251-8 27846799 PMC5111228

[B88] Nigg M. Laroche J. Landry C. R. Bernier L. (2015). RNAseq Analysis Highlights Specific Transcriptome Signatures of Yeast and Mycelial Growth Phases in the Dutch Elm Disease Fungus *Ophiostoma novo-ulmi* . G3: Genes Genomes Genet. 5, 2487–2495. 10.1534/g3.115.021022 PMC4632067 26384770

[B89] Nishida K. Arazoe T. Yachie N. Banno S. Kakimoto M. Tabata M. (2016). Targeted nucleotide editing using hybrid prokaryotic and vertebrate adaptive immune systems. Science 353, aaf8729. 10.1126/science.aaf8729 27492474

[B90] Nishitani C. Hirai N. Komori S. Wada M. Okada K. Osakabe K. (2016). Efficient Genome Editing in Apple Using a CRISPR/Cas9 system. Sci. Rep. 6, 31481. 10.1038/srep31481 27530958 PMC4987624

[B91] Nødvig C. S. Nielsen J. B. Kogle M. E. Mortensen U. H. (2015). A CRISPR-Cas9 System for Genetic Engineering of Filamentous Fungi. PLoS One 10, e0133085. 10.1371/journal.pone.0133085 26177455 PMC4503723

[B92] Nystedt B. Street N. R. Wetterbom A. Zuccolo A. Lin Y.-C. Scofield D. G. (2013). The Norway spruce genome sequence and conifer genome evolution. Nature 497, 579–584. 10.1038/nature12211 23698360

[B93] Pavan S. Jacobsen E. Visser R. G. F. Bai Y. (2009). Loss of susceptibility as a novel breeding strategy for durable and broad-spectrum resistance. Mol. Breed. 25:1. 10.1007/s11032-009-9323-6 20234841 PMC2837247

[B94] Peña L. Séguin A. (2001). Recent advances in the genetic transformation of trees. Trends Biotechnol. 19, 500–506. 10.1016/S0167-7799(01)01815-7 11711193

[B95] Peng A. Chen S. Lei T. Xu L. He Y. Wu L. (2017). Engineering canker-resistant plants through CRISPR/Cas9-targeted editing of the susceptibility gene CsLOB1 promoter in citrus. Plant Biotechnol. J. 15, 1509–1519. 10.1111/pbi.12733 28371200 PMC5698050

[B96] Pennisi E. (2010). Armed and Dangerous. Science 327, 804–805. 10.1126/science.327.5967.804 20150482

[B97] Pourcel C. Salvignol G. Vergnaud G. (2005). CRISPR elements in *Yersinia pestis* acquire new repeats by preferential uptake of bacteriophage DNA, and provide additional tools for evolutionary studies. Microbiology 151, 653–663. 10.1099/mic.0.27437-0 15758212

[B98] Pyott D. E. Sheehan E. Molnar A. (2016). Engineering of CRISPR/Cas9-mediated potyvirus resistance in transgene-free *Arabidopsis* plants. Mol. Plant Pathol. 17, 1276–1288. 10.1111/mpp.12417 27103354 PMC5026172

[B99] Ranjha L. Howard S. M. Cejka P. (2018). Main steps in DNA double-strand break repair: an introduction to homologous recombination and related processes. Chromosoma 127, 187–214. 10.1007/s00412-017-0658-1 29327130

[B100] Richards W. C. (1994). Nonsporulation in the Dutch elm disease fungus *Ophiostoma ulmi*: evidence for control by a single nuclear gene. Can. J. Bot. 72, 461–467. 10.1139/b94-061

[B101] Rizzo D. M. Garbelotto M. (2003). Sudden oak death: endangering California and Oregon forest ecosystems. Front. Ecol. Environ. 1, 197–204. 10.1890/1540-9295(2003)001[0197:SODECA]2.0.CO;2

[B102] Rizzo D. M. Garbelotto M. Davidson J. M. Slaughter G. W. Koike S. T. (2002a). *Phytophthora ramorum* as the Cause of Extensive Mortality of *Quercus* spp. and *Lithocarpus densiflorus* in California. Plant Dis. 86, 205–214. 10.1094/PDIS.2002.86.3.205 30818595

[B103] Rizzo D. M. Garbelotto M. Davidson J. M. Slaughter W. Koike S. T. (2002b). *Phytophthora ramorum* and Sudden Oak Death in California: I. Host Relationships. In: R. B. Stanidord et al. Proceedings of the Fifth Symposium on Oak Woodlands: Oaks in California's Challenging Landscape. (USDA Forest Service Gen. Tech. Rep. PSW-GTR-184), 733–740.

[B104] Roy K. R. Smith J. D. Vonesch S. C. Lin G. Tu C. S. Lederer A. R. (2018). Multiplexed precision genome editing with trackable genomic barcodes in yeast. Nat. Biotechnol. 36, 512–520. 10.1038/nbt.4137 29734294 PMC5990450

[B105] Sbaraini N. Andreis F. C. Thompson C. E. Guedes R. L. M. Junges Â. Campos T. (2017). Genome-Wide Analysis of Secondary Metabolite Gene Clusters in *Ophiostoma ulmi* and *Ophiostoma novo-ulmi* Reveals a Fujikurin-Like Gene Cluster with a Putative Role in Infection. Front. Microbiol. 8, 1063. 10.3389/fmicb.2017.01063 28659888 PMC5468452

[B106] Schuster M. Kahmann R. (2019). CRISPR-Cas9 genome editing approaches in filamentous fungi and oomycetes. Fungal Genet. Biol. 130, 43–53. 10.1016/j.fgb.2019.04.016 31048007

[B107] Schuster M. Schweizer G. Reissmann S. Kahmann R. (2016). Genome editing in *Ustilago maydis* using the CRISPR–Cas system. Fungal Genet. Biol. 89, 3–9. 10.1016/j.fgb.2015.09.001 26365384

[B108] Segretin M. E. Pais M. Franceschetti M. Chaparro-Garcia A. Bos J. II Banfield M. J. (2014). Single Amino Acid Mutations in the Potato Immune Receptor R3a Expand Response to *Phytophthora* Effectors. MPMI 27, 624–637. 10.1094/MPMI-02-14-0040-R 24678835

[B109] Shen Y. Li Y. Xu D. Yang C. Li C. Luo K. (2018). Molecular cloning and characterization of a brassinosteriod biosynthesis-related gene *PtoDWF4* from *Populus tomentosa* . Tree Physiol. 38, 1424–1436. 10.1093/treephys/tpy027 29579304

[B110] Shmakov S. Abudayyeh O. O. Makarova K. S. Wolf Y. II Gootenberg J. S. Semenova E. (2015). Discovery and Functional Characterization of Diverse Class 2 CRISPR-Cas Systems. Mol. Cell 60, 385–397. 10.1016/j.molcel.2015.10.008 26593719 PMC4660269

[B111] Sniezko R. A. Smith J. Liu J.-J. Hamelin R. C. (2014). Genetic Resistance to Fusiform Rust in Southern Pines and White Pine Blister Rust in White Pines—A Contrasting Tale of Two Rust Pathosystems—Current Status and Future Prospects. Forests 5, 2050–2083. 10.3390/f5092050

[B112] Standage-Beier K. Brookhouser N. Balachandran P. Zhang Q. Brafman D. A. Wang X. (2019). RNA-Guided Recombinase-Cas9 Fusion Targets Genomic DNA Deletion and Integration. CRISPR J. 2, 209–222. 10.1089/crispr.2019.0013 31436506 PMC6707420

[B113] Strauss S. H. Tan H. Boerjan W. Sedjo R. (2009). Strangled at birth? Forest biotech and the Convention on Biological Diversity. Nat. Biotechnol. 27, 519–527. 10.1038/nbt0609-519 19513052

[B114] Strauss S. H. Costanza A. Séguin A. (2015). Genetically engineered trees: Paralysis from good intentions. Science 349, 794–795. 10.1126/science.aab0493 26293942

[B115] Strauss S. H. Ma C. Ault K. Klocko A. L. (2016). “Lessons from Two Decades of Field Trials with Genetically Modified Trees in the USA: Biology and Regulatory Compliance,” in Biosafety of Forest Transgenic Trees: Improving the Scientific Basis for Safe Tree Development and Implementation of EU Policy Directives Forestry Sciences. Eds. Vettori C. Gallardo F. Häggman H. Kazana V. Migliacci F. Pilate G. Fladung M. (Dordrecht: Springer Netherlands), 101–124. 10.1007/978-94-017-7531-1_5

[B116] Tanguay P. (2019). CRISPR/Cas9 gene editing of the Dutch elm disease pathogen *Ophiostoma novo-ulmi* . Can. J. Plant Pathol. 41, 163–163. 10.1080/0706066.12019.1519163

[B117] Taylor G. (2002). *Populus*: Arabidopsis for Forestry. Do We Need a Model Tree? Ann. Bot. 90, 681–689. 10.1093/aob/mcf255 12451023 PMC4240366

[B118] Terns M. P. Terns R. M. (2011). CRISPR-based adaptive immune systems. Curr. Opin. Microbiol. 14, 321–327. 10.1016/j.mib.2011.03.005 21531607 PMC3119747

[B119] Toruño T. Y. Stergiopoulos I. Coaker G. (2016). Plant-Pathogen Effectors: Cellular Probes Interfering with Plant Defenses in Spatial and Temporal Manners. Annu. Rev. Phytopathol. 54, 419–441. 10.1146/annurev-phyto-080615-100204 27359369 PMC5283857

[B120] Tsai C.-J. Xue L.-J. (2015). CRISPRing into the woods. GM Crops Food 6, 206–215. 10.1080/21645698.2015.1091553 26357840 PMC5033219

[B121] Tuskan G. A. DiFazio S. Jansson S. Bohlmann J. Grigoriev I. Hellsten U. (2006). The Genome of Black Cottonwood, *Populus trichocarpa* (Torr. & Gray). Science 313, 1596–1604. 10.1126/science.1128691 16973872

[B122] van der Oost J. Jore M. M. Westra E. R. Lundgren M. Brouns S. J. J. (2009). CRISPR-based adaptive and heritable immunity in prokaryotes. Trends Biochem. Sci. 34, 401–407. 10.1016/j.tibs.2009.05.002 19646880

[B123] Wan S. Li C. Ma X. Luo K. (2017). PtrMYB57 contributes to the negative regulation of anthocyanin and proanthocyanidin biosynthesis in poplar. Plant Cell Rep. 36, 1263–1276. 10.1007/s00299-017-2151-y 28523445

[B124] Wang Y. Cheng X. Shan Q. Zhang Y. Liu J. Gao C. (2014). Simultaneous editing of three homoeoalleles in hexaploid bread wheat confers heritable resistance to powdery mildew. Nat. Biotechnol. 32, 947–951. 10.1038/nbt.2969 25038773

[B125] Wang F. Wang C. Liu P. Lei C. Hao W. Gao Y. (2016). Enhanced Rice Blast Resistance by CRISPR/Cas9-Targeted Mutagenesis of the ERF Transcription Factor Gene OsERF922. PLoS One 11, e0154027. 10.1371/journal.pone.0154027 27116122 PMC4846023

[B126] Wang L. Ran L. Hou Y. Tian Q. Li C. Liu R. (2017). The transcription factor MYB115 contributes to the regulation of proanthocyanidin biosynthesis and enhances fungal resistance in poplar. New Phytol. 215, 351–367. 10.1111/nph.14569 28444797

[B127] Wang Q. Cobine P. A. Coleman J. J. (2018). Efficient genome editing in *Fusarium oxysporum* based on CRISPR/Cas9 ribonucleoprotein complexes. Fungal Genet. Biol. 117, 21–29. 10.1016/j.fgb.2018.05.003 29763675 PMC6480338

[B128] Webber J. F. Mullett M. Brasier C. M. (2010). Dieback and mortality of plantation Japanese larch (*Larix kaempferi*) associated with infection by *Phytophthora ramorum* . New Dis. Reps. 22, 19. 10.5197/j.2044-0588.2010.022.019

[B129] Wedge M.-È. Naruzawa E. S. Nigg M. Bernier L. (2016). Diversity in yeast-mycelium dimorphism response of the Dutch elm disease pathogens: the inoculum size effect. Can. J. Microbiol. 62, 525–529. 10.1139/cjm-2015-0795 27068623

[B130] Wiedenheft B. Sternberg S. H. Doudna J. A. (2012). RNA-guided genetic silencing systems in bacteria and archaea. Nature 482, 331–338. 10.1038/nature10886 22337052

[B131] Wullschleger S. D. Tuskan G. A. DiFazio S. P. (2002). Genomics and the tree physiologist. Tree Physiol. 22, 1273–1276. 10.1093/treephys/22.18.1273 12490424

[B132] Xu C. Fu X. Liu R. Guo L. Ran L. Li C. (2017). PtoMYB170 positively regulates lignin deposition during wood formation in poplar and confers drought tolerance in transgenic Arabidopsis. Tree Physiol. 37, 1713–1726. 10.1093/treephys/tpx093 28985414

[B133] Yang L. Zhao X. Ran L. Li C. Fan D. Luo K. (2017). PtoMYB156 is involved in negative regulation of phenylpropanoid metabolism and secondary cell wall biosynthesis during wood formation in poplar. Sci. Rep. 7, 41209. 10.1038/srep41209 28117379 PMC5259741

[B134] Yin T.-M. DiFazio S. P. Gunter L. E. Jawdy S. S. Boerjan W. Tuskan G. A. (2004). Genetic and physical mapping of *Melampsora* rust resistance genes in *Populus* and characterization of linkage disequilibrium and flanking genomic sequence. New Phytol. 164, 95–105. 10.1111/j.1469-8137.2004.01161.x 33873470

[B135] Zhang Y. Ge X. Yang F. Zhang L. Zheng J. Tan X. (2014). Comparison of non-canonical PAMs for CRISPR/Cas9-mediated DNA cleavage in human cells. Sci. Rep. 4, 5405. 10.1038/srep05405 24956376 PMC4066725

[B136] Zhang Y. Bai Y. Wu G. Zou S. Chen Y. Gao C. (2017). Simultaneous modification of three homoeologs of *TaEDR 1* by genome editing enhances powdery mildew resistance in wheat. Plant J. 91, 714–724. 10.1111/tpj.13599 28502081

[B137] Zhang T. Zheng Q. Yi X. An H. Zhao Y. Ma S. (2018). Establishing RNA virus resistance in plants by harnessing CRISPR immune system. Plant Biotechnol. J. 16, 1415–1423. 10.1111/pbi.12881 29327438 PMC6041442

[B138] Zhou X. Jacobs T. B. Xue L.-J. Harding S. A. Tsai C.-J. (2015). Exploiting SNPs for biallelic CRISPR mutations in the outcrossing woody perennial *Populus* reveals 4-coumarate:CoA ligase specificity and redundancy. New Phytol. 208, 298–301. 10.1111/nph.13 25970829

